# Neurological consequences of COVID-19: what have we learned and where do we go from here?

**DOI:** 10.1186/s12974-020-01957-4

**Published:** 2020-09-30

**Authors:** Abbas Jarrahi, Meenakshi Ahluwalia, Hesam Khodadadi, Evila da Silva Lopes Salles, Ravindra Kolhe, David C. Hess, Fernando Vale, Manish Kumar, Babak Baban, Kumar Vaibhav, Krishnan M. Dhandapani

**Affiliations:** 1 Department of Neurosurgery, Medical College of Georgia, Augusta University, 1120 15th Street, 30912 Augusta, Georgia; 2 Department of Pathology, Medical College of Georgia, Augusta University, Augusta, Georgia; 3 Department of Oral Biology and Diagnostic Sciences, Dental College of Georgia, Augusta University, Augusta, Georgia; 4 Department of Neurology, Medical College of Georgia, Augusta University, Augusta, Georgia; 5 Department of Allied Health Science, Shri B. M. Patil Medical College, Hospital and Research Centre, BLDE (Deemed to be University), Vijayapura, Karnataka India

**Keywords:** SARS-CoV-2, ARDS, Neurotropism, Coronavirus, Coagulopathy, Neutrophil extracellular traps, Stroke, Cytokine storm, Neuroinflammation

## Abstract

The coronavirus disease-19 (COVID-19) pandemic is an unprecedented worldwide health crisis. COVID-19 is caused by SARS-CoV-2, a highly infectious pathogen that is genetically similar to SARS-CoV. Similar to other recent coronavirus outbreaks, including SARS and MERS, SARS-CoV-2 infected patients typically present with fever, dry cough, fatigue, and lower respiratory system dysfunction, including high rates of pneumonia and acute respiratory distress syndrome (ARDS); however, a rapidly accumulating set of clinical studies revealed atypical symptoms of COVID-19 that involve neurological signs, including headaches, anosmia, nausea, dysgeusia, damage to respiratory centers, and cerebral infarction. These unexpected findings may provide important clues regarding the pathological sequela of SARS-CoV-2 infection. Moreover, no efficacious therapies or vaccines are currently available, complicating the clinical management of COVID-19 patients and emphasizing the public health need for controlled, hypothesis-driven experimental studies to provide a framework for therapeutic development. In this mini-review, we summarize the current body of literature regarding the central nervous system (CNS) effects of SARS-CoV-2 and discuss several potential targets for therapeutic development to reduce neurological consequences in COVID-19 patients.

## Introduction

A series of pneumonia cases of unknown origin emerged in December 2019 at Wuhan, China, resembling the recent severe acute respiratory syndrome coronavirus (SARS-CoV) and Middle East respiratory syndrome coronavirus (MERS-CoV) outbreaks [1–5]. Genetic sequencing of samples derived from infected patients subsequently identified the pathogen as a novel coronavirus, initially named 2019 novel coronavirus (2019-nCoV), with the associated disease called coronavirus disease-19 (COVID-19) [6]. Given the genetic similarity to SARS-CoV, the nomenclature of the novel coronavirus was later revised to severe acute respiratory syndrome coronavirus 2 (SARS-CoV-2) by the International Committee on Taxonomy of Viruses. Coronaviruses, enveloped positive-sense RNA viruses belonging to family *Coronaviridae* and the order Nidovirales, are widely infectious across species [7]. Indeed, SARS-CoV-2 is believed to have a zoonotic origin and is 96% genetically similar to RaTG13, a previously described bat coronavirus [8]. The highly contagious and virulent nature of SARS-CoV-2 is evidenced by approximately 30,000,000 documented cases and 1,000,000 deaths worldwide, creating a global pandemic that has inflicted economic damage on an unprecedented scale.

The SARS-CoV-2 outbreak has generated immense interest from both the medical community and the general public in understanding the biology, epidemiology, and clinical characteristics, as evidenced by the appearance of over 54,000 peer-reviewed research articles in PubMed focused on COVID-19. SARS-CoV-2 exhibits crossover symptomology with two commonly circulating human coronaviruses (HCoV-NL63, HCoV-HKU1) and prior infection with these strains may greatly impact patient outcomes during the present pandemic. Much of the initial knowledge informing the response to the current SARS-CoV-2 outbreak was gained during the SARS-CoV and MERS-CoV outbreaks. SARS-CoV was first reported in Asia in 2003, spreading to North America, South America, and Europe, which infected over 8000 people worldwide and caused nearly 800 deaths. The subsequent MERS-CoV outbreak was associated with a mortality rate of 37%, with most victims exhibiting one or more comorbid conditions. Likewise, the most characteristic clinical symptoms of COVID-19 patients are fever, fatigue, dry cough, myalgia, headache, dizziness, abdominal pain, diarrhea, nausea, and vomiting. More severe cases involve respiratory distress, which may require admittance to the intensive care unit and use of a ventilator [9]. Patients with underlying comorbidities such as hypertension, diabetes, cardiovascular disorders (CVD), and cerebrovascular diseases are more vulnerable to infection and exhibit a higher rate of hospitalization [9].

**Fig. 1:**
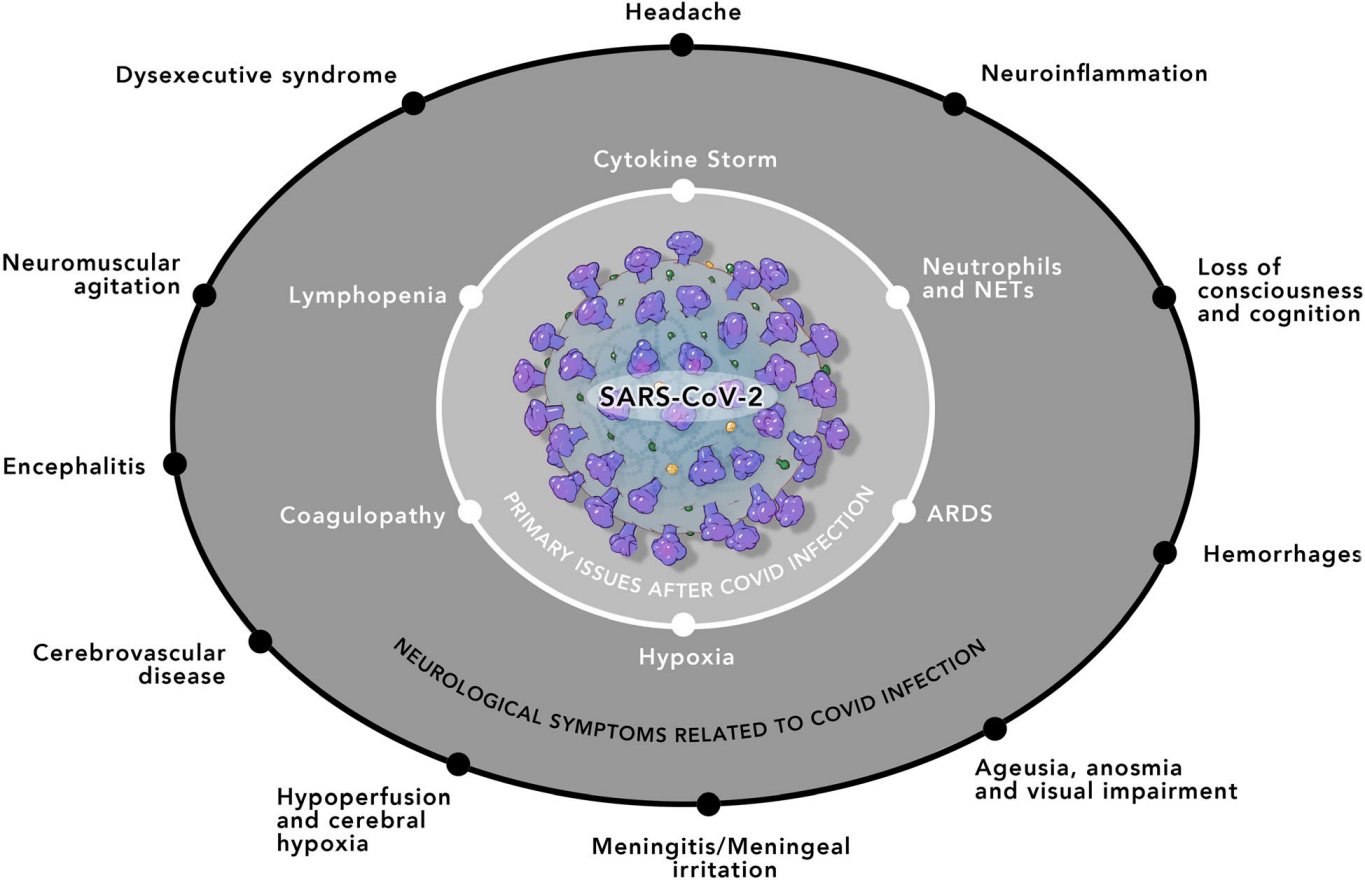
Schematic illustration of COVID-19-related symptoms. Primary issues associated with COVID-19 are shown within the inner circle (see white text). These symptoms are widely reported in a large majority of patients infected with SARS-CoV-2. The outer circle (see black text) depicts neurological issues/symptoms that have been reported after COVID-19

In addition to the classical symptoms of a respiratory virus, increasing evidence suggests COVID-19 patients may present with a diversity of unanticipated neurological symptoms, such as headache, nausea, anosmia, ageusia, myalgia/fatigue, confusion, disorientation, and vomiting [10–12] (Fig. 1). Human coronavirus (HCoV) infections are not restricted to the respiratory tract, with RNA from two HCoV strains (229E, OC43) detected in human brain autopsy samples from neurologically disease patients. Moreover, inter-neuronal propagation and axonal transport may favor viral invasion into the central nervous system (CNS) [11, 13, 14]. Indeed, olfactory and gustatory deficits are regarded as early symptoms of SARS-CoV-2 infection. Of particular interest, reports of ischemic strokes in younger, asymptomatic patients without comorbidities are appearing in the scientific literature, even after the infection has seemingly resolved [15–17]. These limited case reports suggest the need for a deeper understanding of SARS-CoV-2 infection, including elucidation of how the CNS may be affected. Larger clinical studies will undoubtedly shed new light on the clinical manifestations of COVID-19 infection in the brain; however, in this mini-review, we summarize what is currently known regarding SARS-CoV-2-mediated neurological injury to establish a framework for future pre-clinical and clinical investigations. We discuss evidence supporting both hematogenous and retrograde neuronal dissemination of SARS-CoV-2 invasion into the CNS, including secondary neuropathologies, and highlight potential therapeutic approaches for future exploration.

### Neurological manifestations in COVID-19 patients

As COVID-19 rapidly spread throughout the world, anecdotal reports of neurological issues emerged. An internet-based, cross-sectional study found 59 COVID-19 patients from a study population of 1480 patients exhibiting influenza-like symptoms. Notably, loss of smell (68% of COVID-19 patients) and gustatory impairments (71% of COVID-19 patients) were distinguishing features of SARS-CoV-2 infection [10]. In line with this finding, approximately one-third of COVID-19 patients reported a loss of smell. Likewise, headache (about 8%) and nausea and vomiting (1%) were apparent in COVID-19 patients [9, 11, 13]. In addition, case studies of a 24-year-old male infected with SARS-CoV-2 in Japan presented with a fever and meningitis/meningeal irritation [18] while an infected 56-year-old male also was diagnosed with encephalitis [19]. In another case study, a 29-year-old woman diagnosed with COVID-19 presented with a left temporoparietal hemorrhagic venous infarction with transverse sigmoid sinus thrombosis on the left side [20]. These observational reports suggesting CNS involvement in the course of COVID-19 identified many interesting, yet unexplored, avenues for physicians and neuroscientists.

In a study in Strasbourg, France, neurological function was assessed in 58 COVID-19 patients with acute respiratory distress syndrome (ARDS) that were admitted into the intensive care unit (ICU) [12]. Neurological abnormalities were observed in 14% (8/58 patients) upon admission to the ICU, while 67% (39/58) showed neurological signs along with 69% (40/58) who showed agitation following termination of sedation or a neuromuscular blocker [12]. Further, 45% (26/58) of patients showed confusion and corticospinal tract signs were evident in 67% (39/58) of admitted patients. Moreover, 13 patients (22.41%) showing encephalopathic features exhibited leptomeningeal enhancement (8/13) and bilateral frontotemporal hypoperfusion (11/13) on magnetic resonance imaging (MRI). Electroencephalography revealed diffuse bifrontal encephalopathy in one patient (1/8) [12]. Follow-up studies of 45 discharged patients revealed that 33% (15/45) exhibited dysexecutive syndrome and showed signs of inattention, disorientation, and poorly organized movements and response [12].

A study of 214 COVID-19 patients from Wuhan, China, showed severe respiratory infections in 41% (88/214) of patients, with 36% (78/214) of patients displaying diverse neurologic signs, including loss of smell and taste, neuropathic pain, seizures, and strokes [21]. Indeed, loss of smell and taste were similarly reported in COVID-19 patients worldwide [10]. To better understand the neurological manifestations of COVID-19, symptoms were broadly categorized into three categories: skeletal muscular injury indexes, CNS indexes (e.g., acute cerebrovascular disease, headache, dizziness, impaired consciousness, seizure, and ataxia), and peripheral nervous system indexes (nerve pain, impaired taste, smell, or vision). Of the 78 patients displaying neurological abnormalities, 25% showed symptoms related to CNS dysfunction, 11% showed issues related to skeletal muscle injury, and 9% exhibited issues with peripheral nervous system (PNS) function [21]. Of note, neurological symptoms were more commonly observed in older patients (mean age = 59.2 years), in patients with more severe infection, and in patient with pre-existing conditions, such as hypertension, diabetes, malignancy, or cardiac/cerebrovascular disease. Importantly, most of the neurological consequences of COVID-19 were apparent within the first 2 days of infection, although cerebrovascular events and impaired consciousness were often delayed beyond this acute time period and associated with the increased mortality rate [21].

A case study in January 2020, identified a 61-year-old woman that presented with acute weakness in both legs, and severe, progressive fatigue within 1 week after traveling to Wuhan, China. Of note, the observed neurological symptoms and subsequent diagnosis of Guillain-Barre syndrome (GBS) occurred several days prior to the development of respiratory symptoms and before a positive PCR test for SARS-CoV-2 [22]. Similarly, a 67-year-old female patient with a history of breast cancer presented in New York City with rapidly progressive quadriparesis, lower back pain, paresthesias, and urinary retention, diagnosed as severe, rapidly progressing GBS [23]. Moreover, several reports have emerged showing an acute GBS in pediatric patients infected with SARS-CoV-2 [24, 25]. While an association between SARS-CoV-2 infection and symptoms of GBS is evident [26], it remains unclear whether GBS manifestation is a coincidental presentation during SARS-CoV-2 infection or whether this represents a causative relationship.

### How does SARS-CoV-2 directly affect brain function?

The previous section detailed a number of international studies that clearly established the nervous system as a target of COVID-19 infection. These early-stage clinical reports illustrate the need for an improved mechanistic understanding of how SARS-CoV-2 affects neurological function. This knowledge will be essential for the development of efficacious therapies to alleviate suffering in affected individuals. In this section, we propose several mechanisms to explain how a respiratory virus afflicts the CNS.

#### Is SARS-CoV-2 neurotropic?

A simple explanation for the neurological effects of COVID-19 is direct viral entry and infection of the CNS. Epidemiological data show a latency of up to 1 week between the initial infection and hospital admittance for COVID-19 patients [9, 21], providing a window for potential viral entry into the CNS. Neurotropism is commonly observed in coronaviruses, with neuro-invasive properties well documented in SARS-CoV, MERS-CoV, HCoV-229E, HCoV-OC43, and porcine hemagglutinating encephalomyelitis coronavirus (PHE) [11, 13, 27, 28]. The SARS-CoV-2 spike protein also alters barrier function in human models of the blood-brain barrier, providing an additional mechanism of potential CNS entry [29]. Given the genetic similarity and conserved viral structure with SARS-CoV, it appears likely that SARS-CoV-2 may also exhibit neurotropic properties [30, 31].

Tissue distribution of host receptors is generally believed to decide the tropisms of viruses [32–34]. In contrast to MERS-CoV, which exploits dipeptidyl peptidase 4 (DPP4) to evade host cells [35, 36], the densely glycosylated spike protein of SARS-CoV-2 virus binds with high affinity to the type I transmembrane metallocarboxypeptidase, angiotensin-converting enzyme 2 (ACE2), providing a mechanism of viral entry into human cells that mirrors the entry point for SARS-CoV [8, 37–41]. ACE2, which negatively regulates the renin-angiotensin-aldosterone system by degrading angiotensin II to generate angiotensin 1-7, is required to lower blood pressure and as such, is a frequent target for anti-hypertensive drug development [40, 42–46]. Other functions of ACE2 include the metabolism of apelin-13, neurotensin, kinetensin, dynorphin, [des-Arg^9^]-bradykinin, and [Lys-des-Arg^9^]-bradykinin [47]. ACE2 is widely expressed in airway epithelium, lung parenchyma, vasculature, kidney, heart, and the gastrointestinal tract [48, 49], primary sites of infection by SARS-CoV and SARS-CoV-2; however, it is interesting to note that ACE2-expressing endothelial cells and human intestinal cells were unaffected by SARS-CoV [50, 51], while ACE2 negative hepatocytes were susceptible to SARS-CoV infection [32]. Thus, the expression of ACE2 alone may not be sufficient for host cell infection by SARS-CoV-2.

Initial studies failed to observe ACE2 expression in the brain [48, 49]; yet, RT-PCR studies detected low levels of ACE2 mRNA expression in the human brain while subsequent studies revealed that ACE2 immunoreactivity was exclusively within brain endothelial and smooth muscle cells [52]. ACE2 expression is also reported in both neurons and glia [53, 54], suggesting the brain may be a potential target of SARS-CoV-2. Consistent with the possibility of direct CNS infection, SARS-CoV-2 RNA was detectable in the cerebrospinal fluid (CSF), but not in a nasopharyngeal swab from a 24-year-old COVID-19 patient presenting with seizures, hippocampal atrophy, and pan-paranasal sinusitis that was subsequently diagnosed with viral meningitis [18]. Similarly, a 56-year-old encephalitis patient exhibiting reduced consciousness had detectable SARS-CoV-2 in the CSF. These findings in COVID-19 patients are in agreement with reports showing HCoV-OC43 RNA in the CSF of a 15-year-old acute demyelinating encephalomyelitis patient [55], whereas SARS-CoV was detected in the serum and CSF from SARS patients with persistent epilepsy [56]. Therefore, the capacity to leave the respiratory tract and potentially infect other tissues may be a defining feature of CoVs.

#### Does SARS-CoV-2 use a trans-synaptic mechanism of CNS infection?

CoVs may enter the CNS via retrograde neuronal diffusion, potentially via the cribriform plate of the ethmoid bone [57]. In mice, ACE2 and TMPRSS2, a protease that contributes toward the spread of CoVs [58], were expressed in sustentacular cells of the olfactory epithelium, with a more pronounced expression in aged mice [59]. SARS-CoV and MERS-CoV were observed within the CNS, raising the possibility of trans-synaptic viral spread via peripheral nerve terminals as a possible mechanism whereby CoVs may gain access to the CNS [27, 28, 60–62]. SARS-CoV particles were observed in CNS neurons and brain samples from patients diagnosed with SARS [62–64] while other CoVs, including HEV67 and avian bronchitis virus utilized trans-synaptic transfer [27, 28, 61]. Transgenic mice expressing human DPP4 (hDPP4) under the control of the surfactant protein C or cytokeratin-18 promoter showed a progressive fatal course that was paralleled by high viral titers in thalamus and brain stem within 2–6 days after intranasal administration of MERS-CoV [65]. Similarly, lethal intranasal inoculation of SARS-CoV in transgenic mice expressing human ACE2 (hACE2) in the airway and other epithelia resulted in pro-inflammatory activation and the presence of the virus within the olfactory bulb, thalamus, and brain stem via postulated spread through the olfactory nerves [66]. Following the viral entry into the CNS, infection rapidly spread via a trans-neuronal route to other connected brain regions, culminating in mortality due to neuronal loss in the cardiorespiratory centers within the medulla [67]. Finally, SARS was associated with delayed olfactory neuropathy while the loss of olfactory function is an internationally reported symptom of COVID-19, with some patients showing bilateral obstructive inflammation of the olfactory clefts correlating with impaired olfaction [68–71]. Thus, retrograde trans-synaptic transport from the lung and lower respiratory airways to the medullary cardiorespiratory centers of the brain and the olfactory centers may mediate the progressive acute respiratory failure and anosmia in COVID-19 patients.

Beyond trans-synaptic spread from the respiratory system, another possibility is movement via the brain-gut axis. The gastrointestinal (GI) tract is directly infected by SARS-CoV-2 and up to a quarter of COVID-19 patients display GI issues, including nausea, anorexia, vomiting, and diarrhea [72, 73]. A temporal correlation exists between GI and neurological symptoms, and it is postulated that anorexia and nausea may be caused, at least in part, by infection of the lateral hypothalamic nuclei [73, 74]. Toward this end, SARS-CoV-2 may enter the CNS via the vagus nerve, a cranial nerve that regulates parasympathetic control of the heart, lungs, and GI tract.

In addition to direct neuronal entry, SARS-CoV-2 may infect non-neuronal cell types to produce neurological complications. The SARS-CoV-2 entry genes, ACEs and TMPRSS2, are detectable in non-neuronal cell types in the olfactory epithelium and olfactory bulb [75]. Thus, infection of glia and vascular cells could contribute toward hypoperfusion, local inflammation, and cytokine release, loss of function of supporting cells, or damage to sustentacular and Bowman’s gland cells to induce olfactory neuronal dysfunction or death [68, 76–78]. Future studies surely will shed new light on these mechanisms of CNS spread, which may have significant implications for the future treatment of CoV-infected patients.

### Does immune activation contribute to neurological dysfunction after CoV infections?

Inflammation is the first line of defense against pathogens. The innate immune system provides an early mechanism of host protection by producing type I interferons (IFN), complement proteins, and chemokines/cytokines to limit viral infection [79, 80]. While a robust innate immune response is necessary to elicit protective adaptive immunity, a prolonged and/or overactive immune response contributes toward pathological tissue injury [81]. Interestingly, pre-clinical studies showed that excess cytokine release after SARS-CoV infection dampened adaptive immunity [82]. In line with this observation, despite an increase in leukocyte activation and massive release of pro-inflammatory cytokines, SARS-CoV-2 infection is associated with lymphopenia, including suppression of both CD4^+^ and CD8^+^ T cells as well as the increased appearance of exhausted T cells [83–85]. Given this progression, significant attention has been focused on the development of a “cytokine storm,” the rapid pathological release of excess cytokines, which is associated with high fever, respiratory distress, multi-organ failure, and increased mortality over the first 2 weeks in COVID-19 patients [86].

#### Cytokine storm

Critically ill COVID-19 patients exhibited an increased ratio of white blood cells/lymphocytes and higher plasma levels of C-reactive protein (CRP), IL-2, IL-7, IL-10, GSCF, IP10 (CXCL10), MCP-1 (CCL2), MIP-1α (CCL3), and TNF-α, as compared to non-ICU patients [9, 87]. Inflammatory cytokines, such as IL-6, IL-10, and TNF-α, are elevated following infection with SARS-CoV-2 and are believed to orchestrate a cytokine storm [84]. Given these appreciated detrimental effects, a number of clinical trials using tocilizumab, an IL-6 receptor antagonist (NCT04306705, NCT04322773); sarilumab, a IL-6 receptor antagonist (NCT04322773, NCT04315298); or clazakizumab, an IL-6 neutralizing antibody (NCT04343989; NCT04348500), were initiated as potential therapies to limit the cytokine storm in COVID-19 patients.

In contrast to the established association between the cytokine storm and respiratory distress in COVID-19 patients, relatively less is known about the lasting neurological effects of these events. The CNS is regarded as an immune-privileged organ, yet the brain is highly vulnerable to inflammatory mediators and tissue hypoxia [88–91]. Infectious encephalitis is an inflammation of the brain that may develop in bacteria- or virus-infected children, elderly, and immuno-compromised individuals. While mild encephalitis produces transient flu-like symptoms, including fever, headache, seizures, light sensitivity, neck stiffness, and loss of consciousness, more severe cases can produce confusion, psychosis, limb weakness, double vision, cognitive impairments, speech and hearing deficits, coma, and increased fatality. During the course of COVID-19 infection, reports of a rare condition, acute necrotizing hemorrhagic encephalopathy, emerged in patients showing intracranial cytokine storm syndrome without direct viral invasion [92]. Radiological imaging of acute necrotizing hemorrhagic encephalopathy indicates lesions within the thalamus, brain stem, and cerebral white matter [93], suggesting the likely need for neurological assessments of COVID-19 patients. In addition, cytokine-induced pulmonary injury during ARDS may adversely affect brain function due to the intimate association between the lungs and the respiratory centers in the medulla and pons of the brain stem [94–97]. Thus, the neurological manifestations of COVID-19 may be secondary to the consequences of ARDS-mediated inflammation and hypoxemia/hypoxia [94, 95]. As clinical data becomes more widely available regarding the link between the nervous and respiratory systems, this knowledge will greatly shape further pre-clinical efforts.

#### Immunomodulatory therapies to manage the neurological complications from SARS-CoV-2

Understanding the immune dysregulation in patients with COVID-19 will provide a greater understanding of SARS-CoV-2 pathogenesis. The detrimental impact of unrestrained immune activation and the cytokine storm are clearly evident, but therapeutic targets beyond anti-viral drugs remain a major obstacle to limiting neurological injury secondary to COVID-19. As a significant member of the pattern recognition receptor (PRR) family, Toll-like receptors (TLRs) play a crucial role in the initiation of immune responses against viral infections. In addition to initiating the intracellular response to viral RNA, TLRs induce signaling cascades and activate transcription factors that shape the cellular response to infection. Along these lines, activation of TLRs mobilize and recruit innate immune cells (e.g., neutrophils, monocytes, innate lymphoid cells) and induce cytokines and chemokines that limit viral progression and activate acquired immunity [98]. Of the TLRs, TLR3, which is expressed in both immune and non-immune cells, recognizes double-stranded RNA (CoVs are double-stranded RNA viruses). Upon activation, TLR3 induces interferon regulatory transcription factor 3 (IRF3) to stimulate the production of type I interferons as a host defense mechanism against viruses [99]. Importantly, mounting evidence suggests that TLR3 may initiate the cytokine storm and drive systemic inflammatory responses [100–102]. Thus, TLR3 may represent a target for immunotherapeutic modulation to limit neurological dysfunction in COVID-19 patients [103].

#### Do coagulopathies contribute to the neurological consequences of COVID-19?

COVID-19 patients frequently exhibit complications associated with coagulopathy, including venous thromboembolism, acute coronary syndrome, myocardial infarction, and cerebral infarction [104–107]. SARS-CoV-2 infection was associated with prolonged prothrombin time, platelet abnormalities, elevated levels of D-dimer, increased fibrinogen/fibrin degradation products, and sepsis-induced coagulopathy (SIC), a form of disseminated intravascular coagulation (DIC), which was observed in the majority of COVID-19-related deaths [108, 109]. Severe COVID-19 patients exhibit hypoxia, a risk factor that increases thrombosis via activation of hypoxia-inducible transcriptional regulation and by increasing blood viscosity [110]. Given the role of coagulopathy, administration of anticoagulants were postulated as a treatment for severe COVID-19 patients [106, 109]; however, anticoagulation did not reduce life-threatening thrombotic complications in a recent multi-center prospective cohort study of 150 COVID-19 patients with ARDS [105], suggesting the need for extensive research to identify alternative targets for therapeutic intervention.

With respect to the CNS, cytokine release, encephalopathy, and onset of ischemic stroke symptoms are correlated in COVID-19 patients [111, 112]. Inflammation and coagulation are inextricably linked processes that exhibit reciprocal cross-talk [113]. Systemic inflammation activates coagulation mechanisms by driving tissue factor-mediated thrombin generation and inhibiting endogenous fibrinolysis. In turn, activation of the coagulation system may influence inflammatory activity and contribute toward the development of hemorrhagic fever and thrombotic microangiopathy. While a clear association exists between SARS-CoV-2 and stroke incidence, it remains unanswered whether coagulation, secondary to COVID-19 infection, is an initiating factor for ischemic stroke or whether the immune response in response to the viral infection worsens the severity of a stroke. In support of the former possibility, elevated inflammation may heighten the risk of developing an acute ischemic stroke in the elderly, potentially via modulation of the coagulation cascade, whereas the latter possibility may be explained by exacerbation of the post-stroke inflammatory response [114–117]. While it is clear that COVID-19 patients exhibiting pro-thrombotic and/or pro-inflammatory activation may require neurological evaluation, further clinical data and pre-clinical research are needed to define the mechanistic link between SARS-CoV-2 and stroke outcomes.

Given the limited efficacy of broad anticoagulants in COVID-19 patients, alternative therapeutic targets are needed to reduce the detrimental effects of coagulopathies. Neutrophils are circulating innate immune cells that rapidly mobilize to phagocytose pathogens as a mechanism of host protection after an infection. An elevated neutrophil-to-lymphocyte ratio was an independent risk factor for mortality in hospitalized COVID-19 patients [118–120]. Recent evidence suggests that activated neutrophils also may extrude a meshwork of chromatin fibers into the extracellular space to form cloud-like neutrophil extracellular traps (NETs), which may function as a mechanism of pathogen trapping. Extensive infiltration of neutrophils into the pulmonary capillaries of COVID-19 patients was associated with fibrin deposition and vascular lesions in the absence of sepsis while elevated neutrophil counts were associated with ocular dysfunction during SARS-CoV-2 infection [121–125]. Moreover, NETs, which stimulate pro-inflammatory responses in human airway epithelial cells [126], are present in many pulmonary diseases, including asthma, chronic obstructive pulmonary disease (COPD), cystic fibrosis, respiratory syncytial virus bronchiolitis, influenza infection, bacterial pneumonia, ARDS, and tuberculosis [127–132]. While the extent of neutrophil priming and NET formation in ARDS patients correlated with disease severity and mortality [130, 133–136], the clinical significance of NETs in the pathophysiology of COVID-19 remains undefined.

Sera from COVID-19 patients displayed elevated levels of cell-free DNA, myeloperoxidase-DNA complexes, and citrullinated histone H3, suggesting NET formation and raising the possibility that NETs may provide a potential target for intervention in COVID-19 patients [121, 137]. Interestingly, in addition to roles in host defense against viruses and bacteria, NETs also provide a scaffold for thrombogenesis [138, 139]. Indeed, impaired degradation of NETs is clinically associated with acute thrombotic microangiopathies [140], while the presence of citrullinated histone H3, a biomarker of NET formation, within thrombi retrieved from acute ischemic stroke patients was independently associated with patient mortality [141, 142]. Of interest, we recently reported that elevated NET formation was associated with microvascular occlusion and cerebral hypoperfusion after acute brain injury in both mice and humans [143]. Conversely, administration of recombinant human DNase-I, an FDA-approved drug under investigation for the management of COVID-19-induced ARDS [144], improved blood flow and outcomes after both experimental stroke and traumatic brain injury [143, 145–147]. Thus, the widespread generation of NETs after SARS-CoV-2 may provide a potential target to reduce acute and chronic neurological consequences, including headache, elevated stroke risk, and potential cognitive issues due to COVID-19.

### Challenges for the clinical management of COVID-19

A number of medications are being investigated in COVID-19 management, including remdesivir, lopinavir/ritonavir combination, HIV protease inhibitors, chloroquine, and hydroxychloroquine, which may inhibit viral replication in the early stages of infection [148]. In addition, immune-based approaches, such as convalescent plasma, SARS-CoV-2 immunoglobulins, non-specific intravenous immunoglobulins (IVIG), and mesenchymal stem cells, as well as immunomodulatory medications such as corticosteroids (dexamethasone), interferons (IFNα and IFNβ), interleukin inhibitors (IL-1 and IL-6 inhibitors), and kinase inhibitors (Bruton’s tyrosine kinase or Janus kinase inhibitors) are frequently employed as treatment options [3]. On top of the neurological manifestations of SARS-CoV-2, many of these therapies potentially exhibit adverse neurological effects. For example, chloroquine and hydroxychloroquine may be associated with neuropsychiatric adverse effects, retinopathy, ataxia, seizures, and limbic encephalitis [149] while ribavirin and interferons are linked to retinopathy and neuropsychiatric consequences [150]. Seizures a reported symptom of SARS-CoV-2 infection, even in patients with no past medical history of epilepsy; however, an increased occurrence of seizures may be an adverse effect of anti-viral medications (e.g., lopinavir, ritonavir, ribavirin) [151]. Thus, further research to distinguish the deleterious neurological consequences of SARS-CoV-2 from the neurological side effects of COVID-19 therapies is necessary to advance clinical care.

Several co-morbidities associated with neurological dysfunction, including obesity, high body mass index, diabetes, and hypertension correlate with increased rates of infection and worse COVID-19 patient outcomes [152–155]. Therefore, a unique challenge of managing SARS-CoV-2 will be managing the detrimental consequences of co-morbidities with the treatment of COVID-19. Administration of anti-coagulants and statins may encounter drug interactions with the lopinavir/ritonavir combination used for COVID-19 management [150]. Myasthenia gravis or Lambert-Eaton myasthenic syndrome patients receiving immunosuppressive therapy may display a more severe COVID-19 illness and require alternative treatments to avoid myasthenic crisis [156]. In such patients, the administration of IVIG may improve outcomes whereas hydroxychloroquine could worsen the myasthenic crisis [157]. A case report study of a relapsing-remitting multiple sclerosis (MS) patient with SARS-CoV2 infection reported a worsening of neurological symptoms at initial presentation [158]. While the current consensus is to continue disease-modifying treatments, SARS-CoV-2 infected MS patients may benefit from. interferon therapy, suggesting some alterations in the MS treatment regimen may enhance outcomes [159, 160].

Finally, there is a growing appreciation for the psychiatric effects of COVID-19. A comprehensive meta-analysis study of SARS or MERS cases revealed that infected patients exhibited confusion (27.9% of cases), depression (32.6%), anxiety (35.7%), impaired memory (34.1%), and insomnia (41.9%) in the acute phase while post-traumatic stress (32.2%), depression (10.5%), insomnia (12.1%), anxiety (12.3%), irritability (12.8%), and memory impairment (18.9%) chronically persisted after recovery [161]. In line with these findings, COVID-19 patients under intensive care showed signs of delirium with confusion (65%), agitations (69%), and altered consciousness (21%), while 33% showed dysexecutive syndrome at discharge [161]. Therefore, a psychiatric evaluation of patients may be necessary during and beyond hospitalization, including into the chronic term as a possible neurological sequela of COVID-19.

## Conclusions

The COVID-19 pandemic, caused by the novel SARS-CoV-2 virus, is associated with a broad pathophysiology that has resulted in worldwide mortality and morbidity. While primarily regarded as a respiratory virus, SARS-CoV-2 produces wide-ranging and often unpredictable neurological symptoms, ranging from anosmia to encephalitis to increased stroke risk (Fig. 1), that complicate clinical management. Improved development, validation, and implementation of rapid imaging techniques, such as MRI, may aid in early diagnosis and proactive intervention to limit long-term neurological consequences. Future research defining whether SARS-CoV-2 exhibits neurotropism and/or initiates peripheral immune activation and hypercoagulation to affect brain function will be paramount for the development of efficacious therapies to mitigate the deleterious neurological consequences of COVID-19, including potential benefits in the management of acute respiratory failure. Finally, the incorporation of “-omics approaches” will be useful to identify patient populations at the highest risk for developing neurological symptoms. Undoubtedly, biological variables, including sex, age, comorbid conditions (e.g., hypertension, diabetes, stress), pre-existing neurological diseases, and other yet undefined genetic polymorphisms dictate the clinical course of SARS-CoV-2 infection. These unbiased, population-wide investigations will provide valuable information to guide clinical practice in the management of COVID-19, as well as to aid in the management of future pandemics.

## Data Availability

Not applicable.
